# CK-7 negative primary lung adenocarcinoma

**DOI:** 10.1093/jscr/rjad316

**Published:** 2023-06-15

**Authors:** Dimitri Tchienga, Noelani-Mei Ascio, Abid Qureshi, Sultana Razia, Genato Romulo, Armand Asarian, Philip Xiao, Brice Nouthe, Dominique Belinga

**Affiliations:** Department of Surgery, St George’s University School of Medicine, True Blue, Grenada, WI, USA; Department of Surgery, St George’s University School of Medicine, True Blue, Grenada, WI, USA; Department of Surgery, The Brooklyn Hospital Center, Icahn School of Medicine at Mount Sinai, Brooklyn, NY, USA; Department of Surgery, The Brooklyn Hospital Center, Icahn School of Medicine at Mount Sinai, Brooklyn, NY, USA; Department of Surgery, The Brooklyn Hospital Center, Icahn School of Medicine at Mount Sinai, Brooklyn, NY, USA; Department of Surgery, The Brooklyn Hospital Center, Icahn School of Medicine at Mount Sinai, Brooklyn, NY, USA; Department of Pathology, The Brooklyn Hospital Center, Icahn School of Medicine at Mount Sinai, Brooklyn, NY, USA; Fraser Health Authority/Department of Medicine, University of British Columbia, Vancouver, Canada; China Medical University, Shenyang, Liaoning, China

**Keywords:** Lung Adenocarcinoma, cytokeratin, CK7, CK20, TTF1, Napsin A, p40, p63, immunochemistry

## Abstract

Cytokeratin (CK) 7 is normally expressed in the vast majority of lung adenocarcinoma (ADC). However, on rare occasions, as reported in this paper, CK7 negativity can challenge the diagnosis of pulmonary ADC. Hence, the need to use a combination of ‘immunomarkers’ such as thyroid transcription factor 1, Napsin A, p40, p63 and CK20.

## INTRODUCTION

Lung cancer, the most common cause of cancer-related death, is now characterized further by immunohistochemistry that helps identified targeted therapies. This helps for optimal patient selection for specific therapies based on driver mutations. Non-small cell lung cancer (NSCLC) not otherwise specified is now classified into adenocarcinoma (ADC), squamous or large cell carcinoma [1, 2]. The latter is the default nomination when all ‘immunomarkers’ are negative [1]. Squamous carcinoma responds to tumor proteins p40, p63 and cytokeratin (CK) 5/6 [1, 3, 4]. Commonly used markers for ADC are thyroid transcription factor 1 (TTF-1), Napsin A, CK7 and CK AE1/3 [1, 3, 4]. Although the sensitivity and specificity of these markers are variable, CK7 positivity strongly indicates ADC [5, 6]. We are hereby reporting a very rare CK7-negative lung ADC and discussing its implications.

## CASE PRESENTATION

A 60-year-old female with a past medical history of Barrett’s esophagus was admitted with dysphagia to solid foods. The patient reported unintentional weight loss and needed excessive chewing of food and fluids to wash it down. A total of 1 month before admission, the patient’s esophagogastroduodenoscopy showed an 8 cm, near-obstructing, circumferential, ulcerated friable mass extending from the middle third to the lower third of the esophagus. An esophageal biopsy was done, and the pathology report shows Barrett’s esophagus without dysplasia. The computer tomography chest/abdomen/pelvis findings showed a large subcarinal mediastinal mass, likely representing a primary esophageal pathology as described previously. Other findings included right hilar adenopathy and an irregular spiculated lesion in the right lower lobe, which could represent a primary lung malignancy. Biopsy from bronchoscopy and bronchoalveolar lavage revealed a moderately differentiated ADC.

## PATHOLOGY

**Figure 1:**
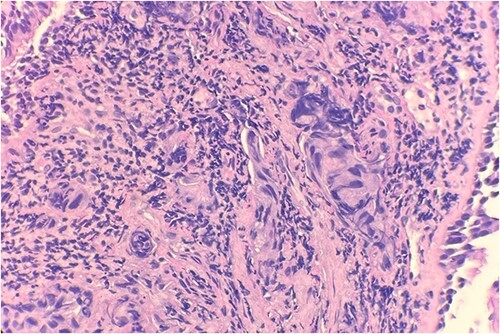
Microscopic examination reveals clusters of tumor cells below the endobronchial lining. Hematoxylin and Eosin (H&E) 40×.

**Figure 2:**
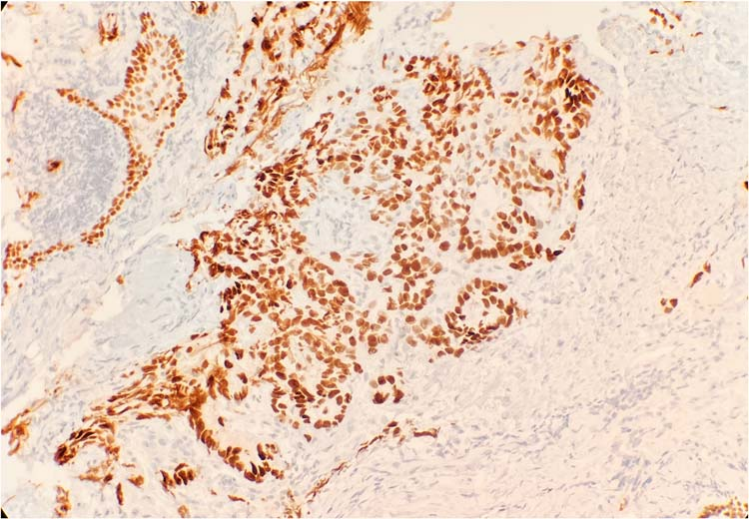
Tumor cells are positive for TTF1 by immunohistochemical stain (IHC) 20×.

**Figure 3:**
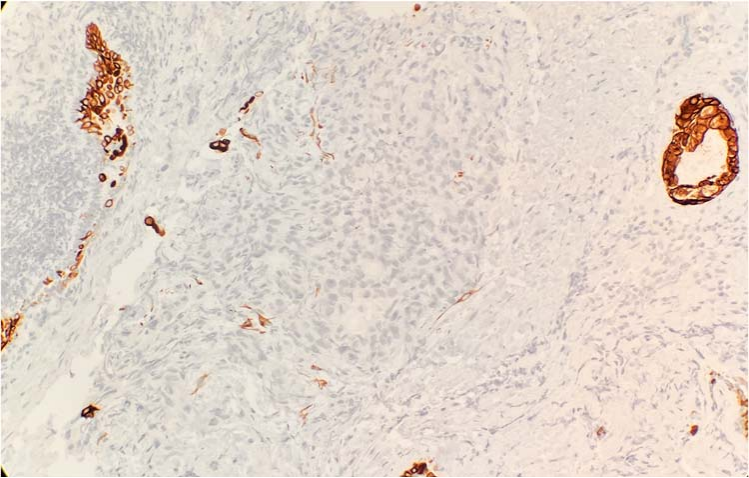
Tumor cells are negative for CK7 by immunohistochemical stain IHC 20×.

Pathology evaluation from the right middle lobe biopsy showed few clusters of malignant cells, consistent with ADC (Fig. 1). Immunohistochemical staining showed tumor cells were positive for AE1/3, Napsin A and TTF1 (Fig. 2), with ⁓60% Ki-67 positivity. Tumor cells were also negative for CK7 (Fig. 3), p40, p63, CK20 and CK5/6. The overall tumor morphology features and immunoprofile favored moderately differentiated ADC of the lung. However, other primary sites could not be ruled out.

## DISCUSSION

The immunohistochemical stains showed positivity for AE1/3, Napsin A, TTF1, 60% Ki-67 and negativity for CK7, CK20, CK5/6, p40 and p63. Gurda *et al*. in their retrospective analysis of 72 patients with primary ADCs, showed that Napsin A sensitivity/specificity of 92.0%/100%, TTF1 showed 84.5%/96.4% and CK7 showed 93.8%/50.0%. In 131 patients with metastatic ADCs, Napsin A showed 67.8%/100%, TTF1 showed 86.9%/87.5% and CK7 showed 100%/25%. The TTF-1/Napsin A combination had an 85.8%/93.4% sensitivity/specificity for primary ADC and 87.8%/88.1% for metastatic ADC. The results of this study, based on Napsin A and TTF1, confirm that our patient’s tumor is an ADC of the lung. CK7 is normally expressed in epithelial lung tissue and CK7 negativity has been used as a criterion to differentiate primary lung ADC (CK7+) with enteric differentiation from metastatic colonic ADC (CK7–) [7, 8]. So far, three cases of primary ADC [3] and two cases of primary ADC with enteric differentiation have been associated with CK7 negativity [7, 8]. According to the International Association for the Study of Lung Cancer, American Thoracic Society and European Respiratory Society, when an NSCLC tumor histology is ambiguous, reclassification could be achieved with either TTF-1 or Napsin A and p63/40 or CK5/6 [1]. P63/40 or CK5/6 will rule out squamous cell carcinoma.

Correct identification and classification of primary or metastatic lung ADC can be achieved by coupling CK7 with other markers such as TTF1 and Napsin A [9]. Current management of primary ADCs includes surgery, chemotherapy, radiation therapy and targeted therapy but none is related to CK7. Hence, its status will not interfere with the treatment approaches. Also, prognostic factors are mainly the depth of tumor invasion and metastasis status, not CK7 status.

There are not enough case reports of CK7 negativity to warrant a reclassification of NSCLCs.

## CONCLUSION

Proper identification of primary ADC can be done using Napsin A+, TTF1 and/or CK7. Though CK7 is normally expressed in lung tissue, in rare cases, CK7 is negative in primary lung ADC. Management and prognosis of ADC should not be affected by CK7 status.

## CONFLICT OF INTEREST STATEMENT

None declared.

## FUNDING

None.
